# *Echinophora tenuifolia* L. subsp. *sibthorpiana* Modulates Stress Response and Mitochondrial Quality Under Glucose Stress in *Caenorhabditis elegans*

**DOI:** 10.3390/antiox15030398

**Published:** 2026-03-21

**Authors:** Monika N. Todorova, Stanislav Dyankov, Martina S. Savova, Velislava Todorova, Milen I. Georgiev, Stanislava Ivanova

**Affiliations:** 1 Laboratory of Metabolomics, Institute of Microbiology, Bulgarian Academy of Sciences, 4000 Plovdiv, Bulgaria; 2 Department of Pharmacognosy and Pharmaceutical Chemistry, Faculty of Pharmacy, Medical University of Plovdiv, 4002 Plovdiv, Bulgaria; 3 Research Institute, Medical University of Plovdiv, 4002 Plovdiv, Bulgaria; 4 Department of Plant Cell Biotechnology, Center of Plant Systems Biology and Biotechnology, 4000 Plovdiv, Bulgaria

**Keywords:** *Echinophora tenuifolia* subsp. *sibthorpiana*, Apiaceae, rutin, *Caenorhabditis elegans*, aging, healthspan, mitochondria, cardiovascular

## Abstract

*Echinophora tenuifolia* L. subsp. *sibthorpiana* (*E*. *tenuifolia*), Apiaceae, is a traditional medicinal and culinary plant, yet its phytochemical composition and biological activity have not been fully investigated. The aim of the present study was to evaluate the chemical profile of *E. tenuifolia* aerial parts extract and to assess its effects on healthspan and metabolic regulation in *Caenorhabditis elegans* (*C*. *elegans*). The characterization of the extract by NMR spectroscopy and HPLC-PDA revealed the presence of secondary metabolites, with rutin being the most abundant phenolic compound identified in the extract, alongside the presence of chlorogenic acid, ferulic acid, rosmarinic acid, caffeic acid, *p*-coumaric acid, and salicylic acid. The extract supplementation enhanced early-life locomotor activity and chemosensory behavior without affecting the lifespan. It also significantly improved thermotolerance and resistance to oxidative stress in *C*. *elegans*. Additionally, in a glucose-induced obesity model, the extract reduced lipid accumulation and triglyceride levels and restored glucose-impaired mitochondrial membrane potential. The extract dose-dependently alleviated glucose-induced endoplasmic reticulum and mitochondrial stress by suppressing the expression of both essential chaperones: endoplasmic reticulum chaperone BiP homolog hsp-4 and heat shock protein hsp-6. These findings indicate that *E. tenuifolia* extract possesses potential beneficial effects on metabolic and mitochondrial health under glucose-induced stress conditions. These observations are likely mediated by the synergistic phenolic composition of the extract, and reveal *E*. *tenuifolia* as a promising source of bioactive compounds relevant to aging and preventive strategies for cardiometabolic health.

## 1. Introduction

In recent decades, pharmacology has increasingly explored plant-derived products and phytochemicals that promote human health and could offer potential protective effects against various acute and chronic conditions [1]. *Echinophora tenuifolia* L. subsp. *sibthorpiana* (Guss.) Tutin (*E*. *tenuifolia*) is a medicinal and aromatic plant of the family Apiaceae. It is distributed across the Eastern Mediterranean, including the Balkans, as well as countries of Southwest and Central Asia, including Turkey and Iran [2]. The plant has a long-established history of medicinal and culinary use in Turkey, where it is employed as an herb, flavoring agent, and natural preservative in various foods [3]. Its incorporation into food products has been reported to improve fermentation processes, nutritional value, and microbiological properties [3,4]. In traditional medicine, the species is utilized for the treatment of a range of medical conditions, including digestive, respiratory and skin disorders [2]. Additionally, phytochemical studies have reported that this species contains a diverse array of compounds, including phenolic acids (e.g., ferulic acid and chlorogenic acid), flavonoids such as rutin and hesperidin [2], and aromatic monoterpenes and phenylpropanoids (e.g., methyl eugenol) [5].

Cardiovascular disorders represent the leading cause of death worldwide [6,7]. These include conditions such as hypertension, atherosclerosis, coronary artery disease, myocardial infarction, arrhythmias, and stroke, all of which are closely interconnected through shared pathophysiological mechanisms. On the other hand, heart failure is a complex clinical syndrome that often develops as the final stage of many of these disorders, reflecting the inability of the heart to pump sufficient blood to meet the body’s metabolic demands [8,9,10]. Importantly, many of these conditions share common disturbances at the cellular and molecular level, including mitochondrial dysfunction, oxidative stress accumulation, and impaired metabolic regulation [6,9,11].

Mitochondria, for example, serve as central regulators of cellular bioenergetics, redox balance, and apoptosis, and their dysfunction has been increasingly recognized as a key contributor to cardiovascular aging and disease [11,12,13,14]. Mitochondrial impairment can lead to increased oxidative stress, reduced adenosine triphosphate (ATP) production, and activation of cell death pathways—all of which contribute to the structural and functional decline of the heart and vascular system [15]. Despite the availability of established therapies for cardiovascular diseases, there is a continuing need to explore complementary or alternative approaches that could enhance prevention and management [6,16].

In the present study, we investigated the phenolic profile of *E*. *tenuifolia* extract by high-performance liquid chromatography with photodiode array detection (HPLC-PDA) and nuclear magnetic resonance (NMR)-based metabolite profiling and evaluated its potential to modulate healthspan and mitochondrial function. Using *C. elegans* as a well-established model for studying conserved mechanisms involved in aging and metabolic regulation, we assessed the extract’s effects on lifespan, thermotolerance, and resistance to oxidative stress, as well as its impact on lipid metabolism and mitochondrial performance under conditions of glucose-induced stress. Through this integrated experimental approach, we aimed to characterize the chemical profile of the extract and further determine whether this traditionally used yet understudied species may represent a valuable source of bioactive compounds capable of modulating healthspan, mitochondrial dysfunction and metabolism homeostasis in *C. elegans*.

## 2. Materials and Methods

### 2.1. Consumables

The reference standards—chlorogenic acid (molecular weight: 354.31 g/M; purity: HPLC ≥ 95%, #89175), caffeic acid (molecular weight: 180.16 g/M; purity: HPLC ≥ 95%, #89547), *p*-coumaric acid (molecular weight: 164.16 g/M; purity: HPLC ≥ 95%, #89498), ferulic acid (molecular weight: 194.19 g/M; purity: HPLC ≥ 95%, #89663), rosmarinic acid (molecular weight: 360.32 g/M; purity: HPLC ≥ 95%, #89266), salicylic acid (molecular weight: 138.12 g/M; purity: HPLC ≥ 95%, #80529), catechin (molecular weight: 290.27 g/M; purity: HPLC ≥ 95%, #89172), rutin (molecular weight: 610.53 g/M; purity: HPLC ≥ 95%, #89270), hesperidin (molecular weight: 610.57 g/M; purity: HPLC ≥ 95%, #89707), quercetin (molecular weight: 302.24 g/M; purity: HPLC ≥ 95%, #89262), luteolin (molecular weight: 286.24 g/M; purity: HPLC ≥ 95%, #89245), kaempferol (molecular weight: 286.24 g/M; purity: HPLC ≥ 95%, #89235), apigenin (molecular weight: 270.24 g/M; purity: HPLC ≥ 95%, #89159), casticin (molecular weight: 374.35 g/M; purity: HPLC ≥ 95%, #89173), and acacetin (molecular weight: 284.27 g/M; purity: HPLC ≥ 95%, #89482)—were obtained from PhytoLab GmbH & Co. KG, Vestenbergsgreuth, Germany. HPLC-grade methanol, acetonitrile, and formic acid were purchased from Merck KGaA (Darmstadt, Germany). Deuterated methanol (CD_3_OD) and water (D_2_O) were supplied from Deutero GmbH (Kasbellaun, Germany).

Nematode Growth Medium (NGM; Cat. No. MBS652667) was obtained from MyBiosource Inc. (San Diego, CA, USA). LB Broth Lennox (Cat. No. L3022), agar powder (Cat. No. 05039), M9 minimal salts (Cat. No. M6030), 3-(4,5-dimethylthiazol-2-yl)-2,5-diphenyl tetrazolium bromide (MTT; Cat. No. M2128), Nile Red (NR; Cat. No. 72485), sodium hydroxide, Fluoroshield histology mounting medium (Cat. No. F6182), paraquat (purity ≥ 98%), 3-(trimethylsilyl)propionic-2,2,3,3-*d*4 acid sodium salt (TSPA-*d*_4_), and Triglyceride Quantification Kit (Cat. No. MAK266) were purchased from Sigma-Aldrich (St. Louis, MO, USA). Tetramethylrhodamine, ethyl ester (TMRE, Cat. No. 11560796), was obtained from Invitrogen (Waltham, MA, USA). MitoView™ Green (MVG, Cat. No. 70054) was supplied by Biotium, Inc. (Fremont, CA, USA).

### 2.2. Plant Material and Extraction

The plant material (aerial parts of *E. tenuifolia* subsp. *sibthorpiana*, including stems, leaves, and inflorescences) was collected in Southeastern Bulgaria (41°50′14.9″ N 26°18′17.3″ E), and a voucher specimen with a voucher number 063306 was deposited in the Herbarium of the Botany and Agrometeorology Department, Agricultural University of Plovdiv (SOA). The air-dried plant was cut, freeze-dried and subsequently ground before extraction. The ground freeze-dried plant material was extracted by ultrasound-assisted extraction with 50% aqueous methanol [17]. After filtration, the extract was concentrated with a rotary vacuum evaporator and further freeze-dried. The dry extract was stored at −20 °C prior to further analyses.

### 2.3. Nuclear Magnetic Resonance (NMR)-Based Metabolite Profiling of E. tenuifolia Extract

Around 50 mg of the dried extract was dissolved in 50% CD_3_OD in D_2_O with TSPA-*d*_4_ as an internal standard according to a previously described procedure [16]. The proton (^1^H NMR) and two-dimensional ^1^H-^1^H homonuclear correlation spectroscopy (COSY) and ^1^H-^13^C heteronuclear single quantum coherence spectroscopy (HSQC) spectra were recorded on an AVII+ 600 spectrometer from Bruker (Karlsruhe, Germany). The obtained data was processed with the MestReNova software, version 12.0.0, from Mestrelab Research (Santiago de Compostela, Spain). The main compounds were identified after comparison with previously published spectral data [18,19,20,21].

### 2.4. HPLC-PDA Analysis

#### 2.4.1. Preparing Standard and Test Solutions

The stock solutions of the analytes (chlorogenic acid, caffeic acid, *p*-coumaric acid, ferulic acid, rosmarinic acid, salicylic acid, catechin, rutin, hesperidin, quercetin, luteolin, kaempferol, apigenin, casticin, and acacetin) were prepared at a concentration of 1 mg/mL with methanol. An ultrasonic bath (Bandelin, Berlin, Germany) was used for better dissolution. Working standard solutions were prepared by serial dilution with water. For the quantification of the analytes in the lyophilized extract, a solution of the extract with a concentration of 1 mg/mL was prepared with water and subsequently diluted according to the concentration range. Before the analysis, the solutions were filtered through a 0.45 µm polytetrafluoroethylene (PTFE) syringe filter (Isolab, Eschau, Germany).

#### 2.4.2. Instrumentation

The HPLC analysis of phenolic compounds was performed using a Shimadzu LC40 system equipped with a photodiode array (PDA) detector SPD-M40 (Shimadzu, Kyoto, Japan). For the separation of the compounds, a Shim-pack C18 (4.6 × 150 mm, 3 μm) column (Shimadzu, Kyoto, Japan) was used.

#### 2.4.3. Chromatographic Conditions

The separation of the compounds was performed using a gradient elution with a constant flow rate of 0.5 mL/min, and a mobile phase comprised of 0.1% formic acid in water (A), methanol (B), and acetonitrile (C). The gradient is presented in Table 1. The column temperature was set at 40 °C. The injection volume was 10 µL. The UV–Vis spectra were recorded in the 190–800 nm range. Each of the chromatograms of the analytes was acquired at absorption maximum as follows: chlorogenic acid—327 nm, caffeic acid—324 nm, *p*-coumaric acid—310 nm, ferulic acid—323 nm, rosmarinic acid—329 nm, salicylic acid—239 nm, catechin—280 nm, rutin—256 nm, hesperidin—284 nm, quercetin—256 nm, luteolin—350 nm, kaempferol—366 nm, apigenin—338 nm, casticin—351 nm, and acacetin—333 nm. The resulting data was analyzed using the LabSolutions software (version 5.118) (Shimadzu, Kyoto, Japan). A system suitability test was carried out according to the United States Pharmacopeia [22], including calculation of separation factor, resolution, number of theoretical plates, and symmetry factor (tailing factor).

#### 2.4.4. Validation of HPLC-PDA Method

After achieving optimal chromatographic conditions and successful separation, the method was validated according to the International Council for Harmonisation of Technical Requirements for the Registration of Medicinal Products for Human Use (ICH) guidelines for linearity, accuracy, precision, limits of detection, limits of quantification, and robustness [23].

##### Linearity, Limit of Detection (LD) and Limit of Quantification (LQ)

The external standard method was employed for the quantification of the selected phenolic acids and flavonoids. Calibration curves were generated using five standard solutions, prepared in concentrations of 5, 10, 25, 50, and 75 µg/mL, and injected in triplicate. The linearity was assessed by correlating the measured areas of the chromatographic peaks with the concentrations of the standard solutions and calculating the coefficient of determination (R^2^). From the linearity data, the linear response and the slope were used for the calculation of the LD and LQ values of each of the analytes.

##### Accuracy and Precision

Evaluation of the accuracy of the developed method was carried out by calculating the percentage recovery, defined as the difference between the mean and the assumed true values. Three quality control concentration levels were selected for each of the analytes as follows: high (50 µg/mL), medium (25 µg/mL), and low (10 µg/mL). The method’s precision was investigated through intra-day repeatability and inter-day reproducibility evaluations. Intra-day precision was assessed by analyzing freshly prepared standard solutions at the three concentration levels described above, with five replicate measurements per level, conducted within a single analytical session. Intra-day precision was assessed by repeating the same protocol over three consecutive days, maintaining the same number of replicates per level.

##### Robustness

The robustness of the method was assessed by varying the column temperature and observing its effect on chromatographic performance. The retention times of the analytes were monitored while intentional temperature changes were applied to the method, with temperature varying between 37 °C and 43 °C. The stability of the standard stock solutions of flavonoids and phenolic acids was evaluated by comparing the chromatograms of the freshly prepared solutions with those of solutions stored at a controlled temperature of 2–8 °C for one week. The temperature changes did not affect the separation of the analytes, and no evidence of degradation or changes in the chemical profiles of the solutions was observed.

### 2.5. Caenorhabditis Elegans Maintenance and Treatment

The Bristol N2 wild-type, SJ4100 zcIs13 [hsp-6p::GFP + lin-15(+)], and SJ4005 zcIs4 [hsp-4::GFP] V strains used in this study were sourced from the Caenorhabditis Genetics Center (CGC) at the University of Minnesota, USA, supported by the NIH Office of Research Infrastructure Programs (P40 OD010440). Worms were cultured under standard laboratory conditions on Nematode Growth Medium (NGM) agar plates, with *Escherichia coli* OP50 provided as a food source. Synchronized worms were obtained by the bleaching method [17]. For experimental treatments, heat-inactivated *E. coli* OP50 was used at a tenfold concentrated final dose. *E. tenuifolia* extract (abbreviated in the figures as ECH) was dissolved in DMSO and administered at final concentrations of 10, 25, and 50 μg/mL (with a final concentration of 0.2%). These concentrations were selected based on a 48 h MTT viability assay to ensure the use of non-toxic doses. The vehicle group, treated with 0.2% DMSO, was used as the control treatment. For analyses of lipid accumulation, mitochondrial function, and GFP reporter strains, glucose was added to the NGM medium to a final concentration of 2% as a model of dysregulated metabolic and mitochondrial homeostasis [24]. All assays were performed in three independent biological replicates.

### 2.6. Locomotion Assay

The locomotion of *E. tenuifolia* extract-supplemented synchronized worms was assessed on both the 5th and 10th days of their lifespan [17]. Briefly, worms were randomly selected and transferred to a drop of M9 buffer, where they were allowed a 30 s acclimation period. The body bends within a 30 s interval were then recorded using a KERN & SOHN GmbH (Balingen, Germany) stereomicroscope. The assay was performed in three independent biological replicates, with at least 15 worms per experimental group.

### 2.7. Lifespan Measurement

Synchronized late-L4-stage larvae (30 worms per group) were transferred to NGM plates containing either *E. tenuifolia* extract (10, 25, and 50 μg/mL) or vehicle. This was defined as day 0 of their lifespan. Worms were monitored daily for survival, and deceased individuals were recorded until all worms had died. Regular transfers to fresh plates were conducted every 2–3 days. The assay was performed in three independent biological replicates. The obtained data were pooled and represented as Kaplan–Meier survival curves [17].

### 2.8. Chemotaxis Assessment

For the chemotaxis assay, a Petri dish was divided into four quadrants, with one or two quadrants designated for test samples and the remaining for controls [25]. A 2 μL volume of each treatment was added to the respective quadrants. Approximately 150 synchronized L4 nematodes were placed at the centre of the dish. Following a 1 h incubation at 20 °C, the dish was cooled to 4–6 °C for 30 min to immobilize the nematodes. Worms in each quadrant were then counted, and the chemotaxis index (CI) was calculated using the formula CI = (quadrant test area 1 + quadrant test area 2) − (quadrant control area 1 + quadrant control area 2)/total number of nematodes.

### 2.9. Nile Red Staining and Triglycerides Quantification

Following a 24 h treatment with *E. tenuifolia* extract, approximately 1500 age-synchronized L4 larvae per experimental group were collected. Staining was performed as previously described using Nile Red dye [24,25]. Imaging was conducted with a Stellaris 5 confocal system equipped with an inverted DMi8 microscope (Leica, Wetzlar, Germany). Microphotographs for all biological replicates were captured under identical image acquisition settings. Fluorescence intensity was quantified using the ImageJ software; background subtraction and the following representation of the results, as normalized corrected total cell fluorescence (CTCF) in arbitrary units (a.u.), were performed according to the previously described procedure [26].

Measurement of triglyceride content was performed using the Triglyceride Quantification Kit (MAK266, Sigma-Aldrich, St. Louis, MO, USA). Sample preparation, measurement, and data calculations were carried out according to the manufacturer’s protocol. The obtained triglyceride values (nM) for each treatment group were normalized to the vehicle (+G) group and presented in arbitrary units (a.u.).

### 2.10. Thermotolerance Assay

Heat stress was induced by incubating age-synchronized worms at 37 °C for 2 h, followed by a 20 h recovery period [17]. After the recovery period, survival was assessed, with worms being considered dead if they failed to respond to a gentle touch with a platinum wire. Each experimental group consisted of at least 30 nematodes, and the experiments were independently repeated three times.

### 2.11. Oxidative Stress Assay

For the oxidative stress assay, paraquat was used as a well-known herbicide that induces oxidative stress by generating ROS. Age-synchronized nematodes pre-treated with either *E. tenuifolia* extract or vehicle were transferred to fresh NGM plates containing 50 mM paraquat on the 5th and 10th days of their lifespan [27]. Survival was monitored at 24 h intervals until the death of the last worm. Each experimental group included a minimum of 30 nematodes per biological replicate, and the experiments were performed in three independent biological replicates.

### 2.12. Confocal Imaging of Transgenic Reporter Strains

To assess the mitochondrial unfolded protein response (UPRmt) and endoplasmic reticulum (ER) stress under conditions of excessive glucose and *E. tenuifolia* extract supplementation, the following reporter strains were used: the mitochondrial Hsp70 chaperone *hsp-6* (SJ4100) and the BiP homologue *hsp-4* (SJ4005). Age-synchronized worms from each strain and experimental group were pre-treated with the extract for 24 h, collected, washed with M9 buffer, and mounted onto microscope slides. For the SJ4005 strain, a brief heat stress was used (37 °C for 5 min) as a positive control group for *hsp-4* expression. All experiments were performed in at least three independent biological replicates. Imaging was conducted using a Stellaris 5 confocal system coupled with an inverted DMi8 microscope (Leica, Wetzlar, Germany). Quantification of fluorescence intensity was carried out using the ImageJ software (version 1.53t) [17,24].

### 2.13. Mitochondrial Staining

To measure mitochondrial function, including mitochondrial membrane potential (_Δ_Ψm) and mitochondrial mass in conditions of glucose-induced mitochondrial stress, co-staining with the mitochondrial dyes TMRE and MVG was performed [17,24]. Both dyes were added directly to the experimental treatments comprising *E. tenuifolia* extract (10, 25, and 50 μg/mL) at final concentrations of 100 nM for TMRE and 4 μM for MVG. Synchronized L4-stage worms (approximately 500 worms per experimental group) were transferred onto NGM plates containing *E. tenuifolia* extract and the mitochondrial dyes and incubated for 24 h. After incubation, worms were transferred to treatment-free plates for a brief washout period to remove residual dye from the intestine. Imaging was performed using a Stellaris 5 confocal system coupled with an inverted DMi8 microscope (Leica, Wetzlar, Germany). Quantification of fluorescence intensity was carried out using the ImageJ software (version 1.53t) [17,24]. All experiments were performed in at least three independent biological replicates.

### 2.14. Statistical Analysis

Statistical analyses were performed in SigmaPlot v11.0 from Systat Software GmbH (Erkrath, Germany). Data are represented as mean ± SEM. Differences among the experimental groups were analyzed by one-way ANOVA, followed by Tukey’s post hoc test. Statistical significance was set at *p* < 0.05 and *p* < 0.01. Comparisons with vehicle without glucose (−G) are denoted by “*”, whereas comparisons with glucose-supplemented vehicle (+G) are indicated by “#”. When data failed to meet the normality assumption, ANOVA on ranks (Kruskal–Wallis test) was applied for multiple comparisons. For lifespan and oxidative stress, Kaplan–Meier survival curves were compared using the log-rank test. The experimental data presented are representative of at least three independent biological experiments.

## 3. Results

### 3.1. Metabolite Profiling of E. tenuifolia Extract Based on NMR Spectroscopy

**Figure 1:**
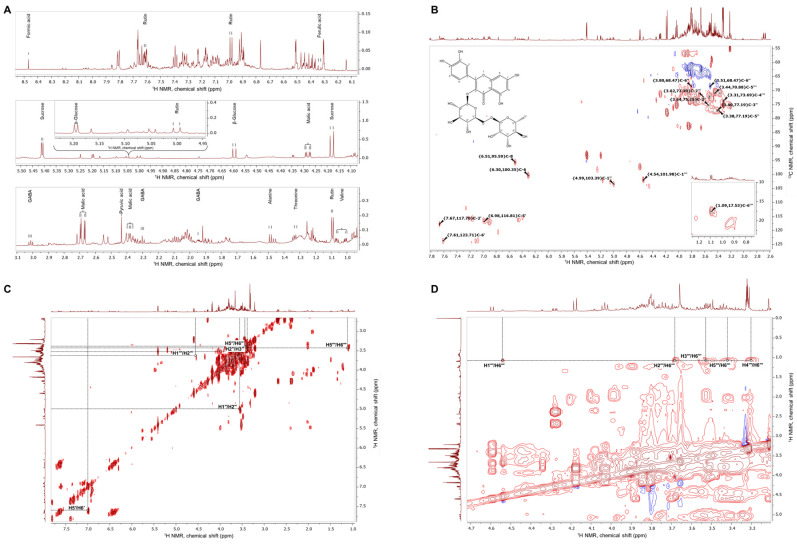
Representative NMR spectra of *E. tenuifolia* aerial parts extract. (**A**) Assignments of identified primary and secondary metabolites in the proton NMR spectra zoomed-in regions for *δ* 1–3.1 ppm, *δ* 4.1–5.5 ppm, and *δ* 6.1–8.5 ppm. (**B**) ^1^H-^13^C heteronuclear single quantum coherence spectrum (HSQC) with annotated characteristic signals of rutin (molecular weight, 610.517 g/M; IUPAC name, 2-(3,4-dihydroxyphenyl)-5,7-dihydroxy-3-[(2S,3R,4S,5S,6R)-3,4,5-trihydroxy-6-[[(2R,3R,4R,5R,6S)-3,4,5-trihydroxy-6-methyloxan-2-yl]oxymethyl]oxan-2-yl]oxychromen-4-one). Red signals represent CH or CH_3_ groups, while blue signals represent CH_2_ groups. (**C**) ^1^H–^1^H homonuclear correlation spectrum (COSY) and (**D**) zoomed-in ^1^H–^1^H TOCSY spectra with the assignments of spin system signals from rhamnosyl and glucosyl moieties in the rutin structure.

The extract of *E*. *tenuifolia* was subjected to metabolite profiling by NMR. Analysis of the proton NMR spectra of *E. tenuifolia* extract revealed the presence of common primary metabolites, comparing the obtained spectra with spectral data from the literature [18,20,21]. Corresponding chemical shifts (*δ*, ppm) and coupling constants (*J*, Hz) of the identified metabolites are listed in Supplementary Table S1. Among the specialized metabolites, within the proton spectra were assigned some signals of phenolic compounds—ferulic acid and rutin (Figure 1A, Supplementary Table S1).

To further confirm the presence of the most abundant secondary metabolites, analysis of the acquired two-dimensional ^1^H-^1^H-COSY, ^1^H-^1^H-TOCSY and ^1^H-^13^C-HSQC was performed. Compared with the literature data [19], the cross-peaks between corresponding carbon and hydrogen atoms within the rutin structure were assigned to the *E. tenuifolia* extract HSQC spectra (Figure 1B) and the proton signals—listed in Supplementary Table S1. The correlation within the COSY (Figure 1C) and TOCSY spectra (Figure 1D) suggested the presence of a characteristic spin system of the rutin disaccharide moiety, in accordance with previously reported observations [28].

### 3.2. Chromatography Analysis

An HPLC-PDA method was developed for the quantification of some commonly found phenolic compounds in plants, including the phenolic acids chlorogenic acid, caffeic acid, *p*-coumaric acid, ferulic acid, rosmarinic acid, and salicylic acid, as well as the flavonoids catechin, rutin, hesperidin, quercetin, luteolin, kaempferol, apigenin, casticin, and acacetin. Well-resolved chromatographic peaks (Figure S1) that demonstrated good symmetry and peak shape were achieved with the mobile phase, as described in Section 2.4.3. System suitability parameters (separation factor, resolution, theoretical plates, and tailing factor) confirmed the efficient separation (Table S2), and the method was subsequently validated. The method proved accurate, precise, linear, robust, and suitable for the determination of phenolic acids and flavonoids. The acquired data for linearity and the determined values for limit of detection and limit of quantification are presented in Supplementary Materials, Table S3, while a summary of the accuracy and precision evaluations is presented in Table S4 and Table S5, respectively.

The method was applied to identify and quantify the selected phenolic acids and flavonoids in the extract. Six phenolic acids were identified in the *E*. *tenuifolia* extract, including chlorogenic acid, *p*-coumaric acid, ferulic acid, rosmarinic acid, salicylic acid, and caffeic acid, as well as the flavonoid rutin. The compounds were quantified through the calibration curves, and it was determined that rutin was the most abundant compound identified in the extract (43.86 mg/g dry extract). The calculated contents of chlorogenic acid, *p*-coumaric acid, ferulic acid, rosmarinic acid, salicylic acid, and caffeic acid were 5.50 mg/g, 0.41 mg/g, 5.12 mg/g, 4.29 mg/g, 2.10 mg/g, and 0.98 mg/g dry extract respectively. All measurements were performed in triplicate, with a standard deviation not exceeding 2%.

### 3.3. The E. tenuifolia Extract Modulates the Chemosensory Network of C. elegans and Increases Early-Life Energy Expenditure

**Figure 2:**
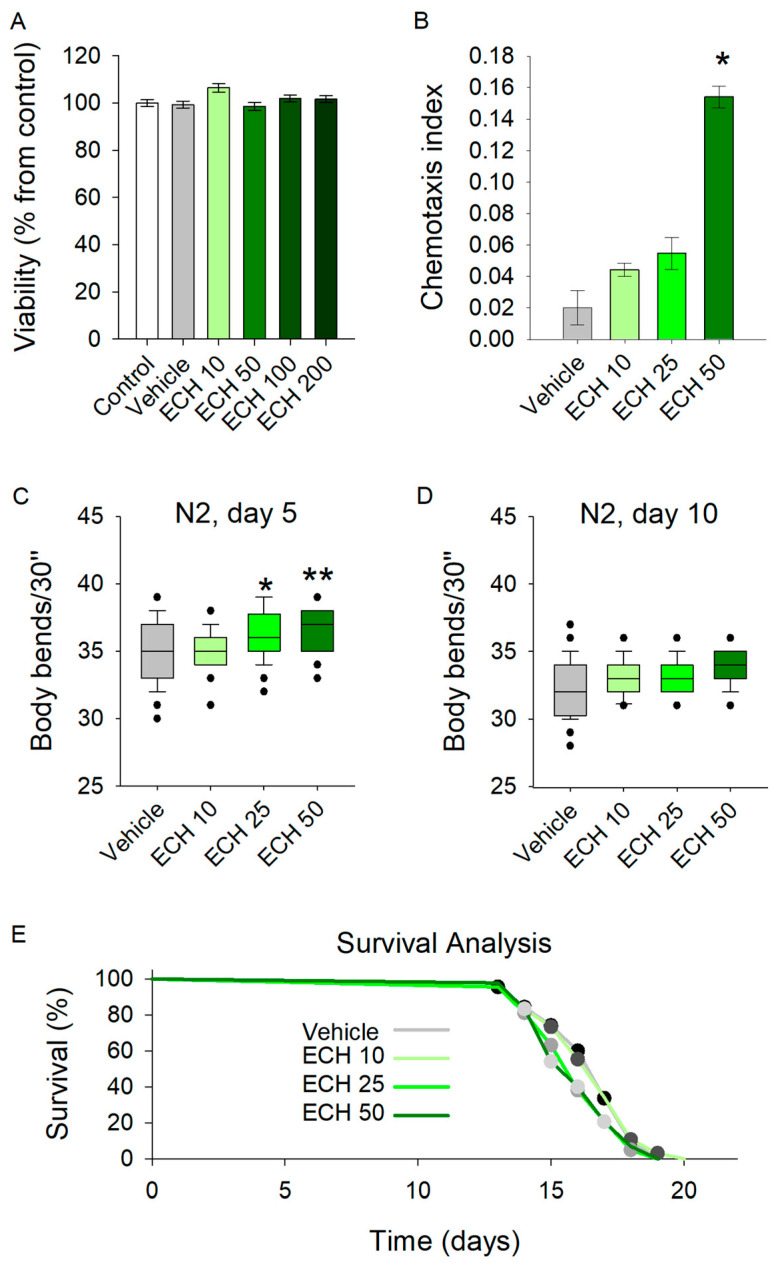
The *E. tenuifolia* extract (ECH) enhances locomotion and modulates the chemosensory network in *C. elegans*. (**A**) Viability test upon treatment with 10–200 μg/mL *E. tenuifolia* extract in *C. elegans* (*n* = 1500), using ANOVA on ranks vs. control group. (**B**) Chemotaxis analysis on worms pre-treated with the *E. tenuifolia* extract (*n* = 300–600), t-test vs. vehicle. (**C**,**D**) Locomotion of pre-treated worms (10, 25 and 50 μg/mL *E. tenuifolia* extract) for 5 and 10 days. Results are presented as bending movements within 30 s and compared to vehicle group (*n* = 45) in one-way ANOVA. (**E**) Lifespan of treated worms (10, 25 and 50 μg/mL *E. tenuifolia* extract) represented as Kaplan–Meier survival curve (*n* = 90). Log-rank test was used to evaluate the statistical significance between experimental treatment survival curves, ** *p* < 0.01, * *p* < 0.05.

By evaluating the safety profile of *E. tenuifolia* extract across a range of concentrations (10 to 200 μg/mL) for 48 h, the results demonstrated no adverse effects on viability in any treatment group (Figure 2A). Therefore, concentrations of 10, 25, and 50 μg/mL were selected for further experiments.

The chemotaxis assay revealed a significant enhancement in sensory-driven behavior at the highest tested concentration of 50 μg/mL (Figure 2B). Worms exposed to *E. tenuifolia* extract at this dose exhibited improved responses to sensory cues, suggesting that the extract may act as an attractant or modulate the chemosensory signaling pathways in *C. elegans*. Interestingly, lower concentrations (10 and 25 μg/mL) did not yield a comparable effect.

Exposure to all tested concentrations of the extract significantly increased the body bends of 5-day-old *C. elegans* (Figure 2C). In contrast, the extract did not influence locomotion in 10-day-old worms (Figure 2D). These findings suggest that *E. tenuifolia* supplementation may enhance energy expenditure and exert a stimulatory effect on the nervous system in young worms. However, the absence of any observed effect in aged worms indicates that *E. tenuifolia* extract may not provide benefits related to aging.

To assess the potential role of the extract in longevity, we tested its effect on the lifespan of *C. elegans*. The analysis revealed no significant differences in lifespan across any tested concentrations (10, 25, and 50 μg/mL) compared to the control group (Figure 2E). These results suggest that *E. tenuifolia* extract does not exert a measurable influence on lifespan under the tested conditions.

### 3.4. The E. tenuifolia Extract Has Antioxidant Capacity and Increases Survival to Acute Heat Stress

Thermotolerance is a frequently studied marker in aging research, as increased lifespan often correlates with improved stress resilience across different model systems [17,29]. Moreover, in age-related diseases such as cardiovascular and neurodegenerative disorders, the inability of the organism to adequately manage stress conditions leads to the accumulation of misfolded proteins and disrupted proteostasis [30,31,32]. To evaluate the stress-resistance potential of *E. tenuifolia* extract, *C. elegans* was pre-treated with 10, 25, and 50 μg/mL of the extract for 5 and 10 days before being exposed to 37 °C for 2 h.

**Figure 3:**
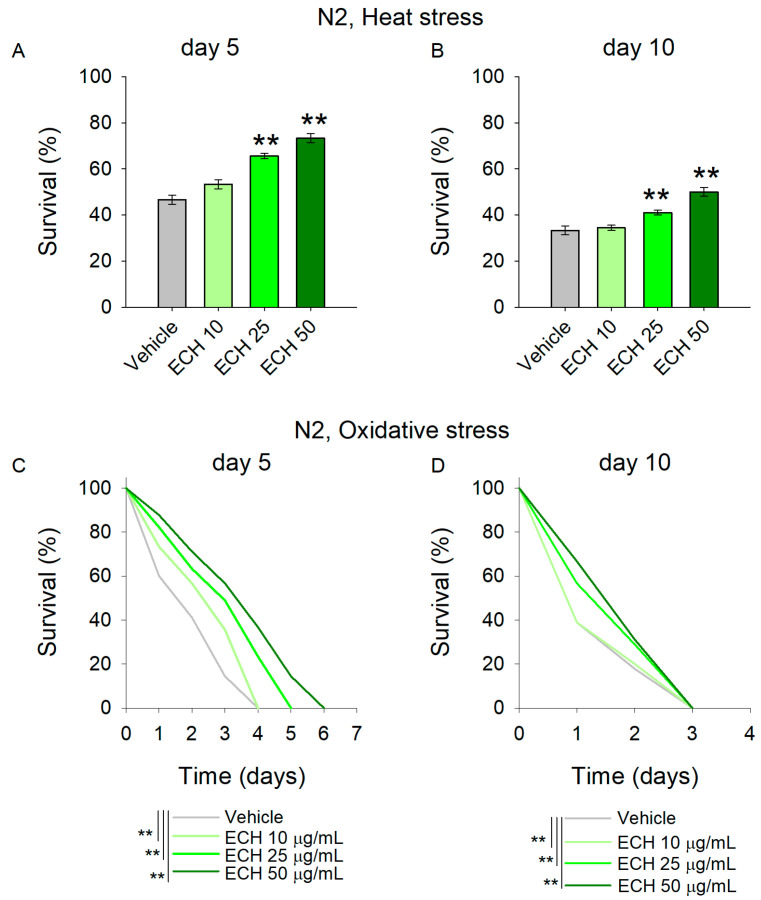
The *E*. *tenuifolia* extract (ECH) supplementation modulates stress response in *C*. *elegans*. (**A**,**B**) To assess thermotolerance, (**A**) 5-day-old and (**B**) 10-day-old worms pre-treated with *E. tenuifolia* extract were exposed to acute heat stress (37 °C for 2 h). Following a 20 h recovery period, survival was recorded. Data are presented as mean percentage survival ± SEM (*n* = 90). Statistical significance: ** *p* < 0.01 (one-way ANOVA). (**C**,**D**) To evaluate oxidative stress resistance, worms pre-treated with *E. tenuifolia* extract were exposed to 50 mM paraquat. Survival was monitored at 24 h intervals in both (**C**) 5-day-old and (**D**) 10-day-old worms. Data for oxidative stress survival are presented as Kaplan–Meier survival curve (*n* = 90). Log-rank test was used to evaluate the statistical significance between experimental treatment survival curves, ** *p* < 0.01.

The results revealed that worms supplemented with *E. tenuifolia* demonstrated improved thermotolerance compared to the vehicle group (Figure 3A,B). Specifically, concentrations of 25 and 50 μg/mL significantly enhanced stress resistance at both time points. These findings suggest that the extract may interact with the stress-response network, potentially supporting repair processes following heat exposure, even in aged worms.

Along with the accumulation of damaged proteins, antioxidant defense mechanisms are often compromised in cardiovascular diseases, leading to excessive oxidative stress and frequently to mitochondrial dysfunction [33,34]. Paraquat, a commonly used herbicide, is widely employed as a reliable inducer of oxidative stress in *C*. *elegans* studies [27,35]. Acute paraquat exposure initiates a cascade of biochemical reactions that culminate in the disruption of cellular redox homeostasis. In this study, pre-treatment with the extract significantly mitigated the detrimental effects of 50 mM paraquat in young worms (Figure 3C), indicating enhanced resistance to acute oxidative stress. In contrast, *E*. *tenuifolia* extract supplementation did not improve the survival of aged worms challenged with paraquat (Figure 3D; Supplementary Table S6), suggesting an age-dependent decline in the extract’s protective capacity with respect to oxidative stress-induced damage.

### 3.5. The Extract of E. tenuifolia Reduces Lipid Accumulation in a Glucose-Induced Obesity Model in C. elegans

Excessive fat is a key driver of low-grade inflammation, insulin resistance, and altered lipid metabolism [36,37,38], all of which contribute to the progression of cardiovascular diseases [36,39]. Given the complex composition of the extract and the presence of key bioactive compounds such as chlorogenic acid, caffeic acid, and rosmarinic acid—each with well-known biological activities related to obesity and lipid metabolism regulation [40,41,42,43]—we reasoned that the extract may influence metabolic regulation. To explore this possibility, we tested the effect of *E. tenuifolia* extract on lipid accumulation in a glucose-supplemented obesity model in *C. elegans,* alongside triglyceride quantification.

**Figure 4:**
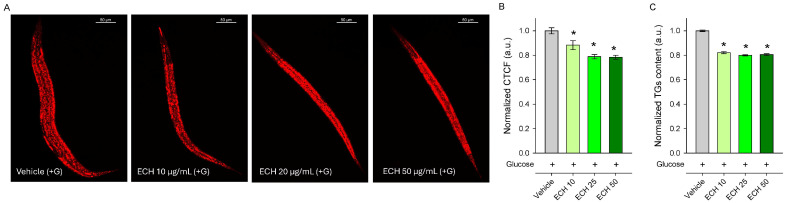
The *E. tenuifolia* extract (ECH) modulates lipid metabolism in *C. elegans* obesity model. (**A**) Representative confocal images at 20× magnification (scale bar: 50 μm) of Nile Red-stained lipids in glucose-supplemented (+G) wild-type N2 nematodes treated for 24 h with *E. tenuifolia* extract at concentrations of 10, 25, and 50 μg/mL compared to the vehicle (+G). (**B**) Quantification of fluorescence intensity of Nile Red lipid staining was represented as corrected total cell fluorescence (CTCF), normalized to vehicle (+G), expressed in arbitrary units (a.u.). (**C**) Triglyceride content for *E. tenuifolia*-treated groups was normalized to vehicle (+G) and expressed in a.u. Results are presented as mean ± SEM (*n* = 90), * *p* < 0.05 compared to the vehicle group (ANOVA on ranks).

In agreement with our expectations, worms pre-treated with the extract for 24 h exhibited a significant reduction in lipid content compared to the vehicle-treated group, as demonstrated by both Nile Red staining (Figure 4A,B) and triglyceride quantification (Figure 4C). Although Nile Red staining is widely used to visualize lipid stores in *C. elegans*, it is worth mentioning that this method is semi-quantitative and may be influenced by factors such as feeding behavior and intestinal physiology. Therefore, triglyceride quantification provides a more direct assessment of lipid content and supports the observed reduction in fat accumulation. These findings also correspond with the traditional culinary use of the plant as a spice in Mediterranean and Middle Eastern cuisine. Overall, the results indicate that *E. tenuifolia* extract modulates lipid metabolism and may counteract excessive fat storage under glucose-rich conditions.

### 3.6. The Extract of E. tenuifolia Restores Mitochondrial Membrane Potential in a Model of Glucose-Induced Mitochondrial Impairment

**Figure 5:**
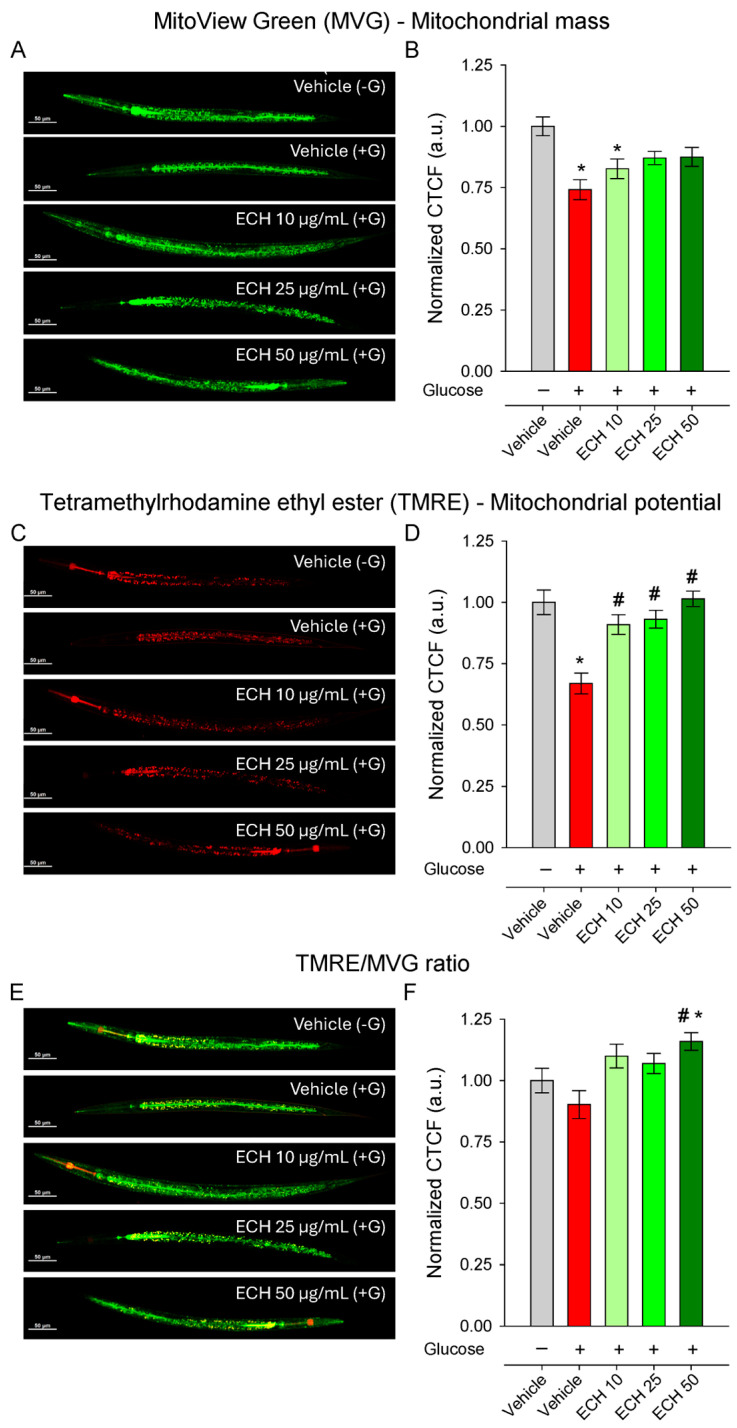
The extract of *E. tenuifolia* (ECH) restores mitochondrial potential under glucose stress. Representative confocal images and CTCF normalized to the vehicle (−G) of (**A**,**B**) MVG, (**C**,**D**) TMRE-stained N2 worms and (**E**,**F**) ratio of TMRE to MVG whole-body fluorescence. Worms were treated for 24 h with 10, 25, or 50 μg/mL *E*. *tenuifolia* extract or vehicle, either with (+G) or without glucose (−G). Mean ± SEM, *n* = 60; * *p* < 0.05 compared to vehicle (−G), and # *p* < 0.05 compared to vehicle (+G); ANOVA on ranks.

Mitochondria serve as central regulators of cellular bioenergetics, redox balance, and apoptosis, and their dysfunction has been increasingly recognized as a key contributor to cardiovascular aging [11,44]. To determine whether *E. tenuifolia* extract supplementation counteracts glucose-induced mitochondrial stress, we quantified mitochondrial membrane potential (_Δ_Ψm) and mitochondrial mass using TMRE and MVG staining, respectively. Exposure to 2% glucose significantly reduced both _Δ_Ψm and mitochondrial mass (Figure 5A–D) compared to the control group, although the TMRE/MVG ratio remained unchanged (Figure 5E,F), indicating a proportional decline in mitochondrial function and content.

The extract treatment restored mitochondrial membrane potential in a dose-dependent and statistically significant manner (Figure 5E,F), with the highest concentration returning _Δ_Ψm to levels comparable to the vehicle (−G) conditions. In contrast, mitochondrial mass remained unaffected by *E. tenuifolia* extract at all tested concentrations (Figure 5C,D), suggesting that the extract primarily enhances mitochondrial functional quality rather than altering mitochondrial abundance.

Interestingly, the TMRE/MVG ratio was significantly increased only at the highest *E. tenuifolia* extract concentration (50 μg/mL; Figure 5E,F), both compared to vehicle +G and to vehicle −G, suggesting an improvement in mitochondrial efficiency or respiratory competence per mitochondrial unit.

### 3.7. The Extract of E. tenuifolia Attenuates Glucose-Induced Endoplasmic Reticulum and Mitochondrial Stress by Regulating hsp-4 and hsp-6 Expression

When mitochondrial integrity is compromised, cells activate protective mechanisms such as the mitochondrial unfolded protein response (UPRmt), which operates in close cooperation with endoplasmic reticulum (ER) stress pathways to restore protein folding capacity and maintain cellular function [45,46]. Taking into account that *E. tenuifolia* supplementation improved thermotolerance in both young and aged worms—suggesting a potential modulation of the proteostatic network—and considering the central crosstalk between UPRmt and ER stress in mitochondrial quality control [47,48], we examined whether *E. tenuifolia* extract could influence these two pathways by assessing the expression of the mitochondrial chaperone *hsp-6* (UPRmt) and the ER stress marker *hsp-4*.

**Figure 6:**
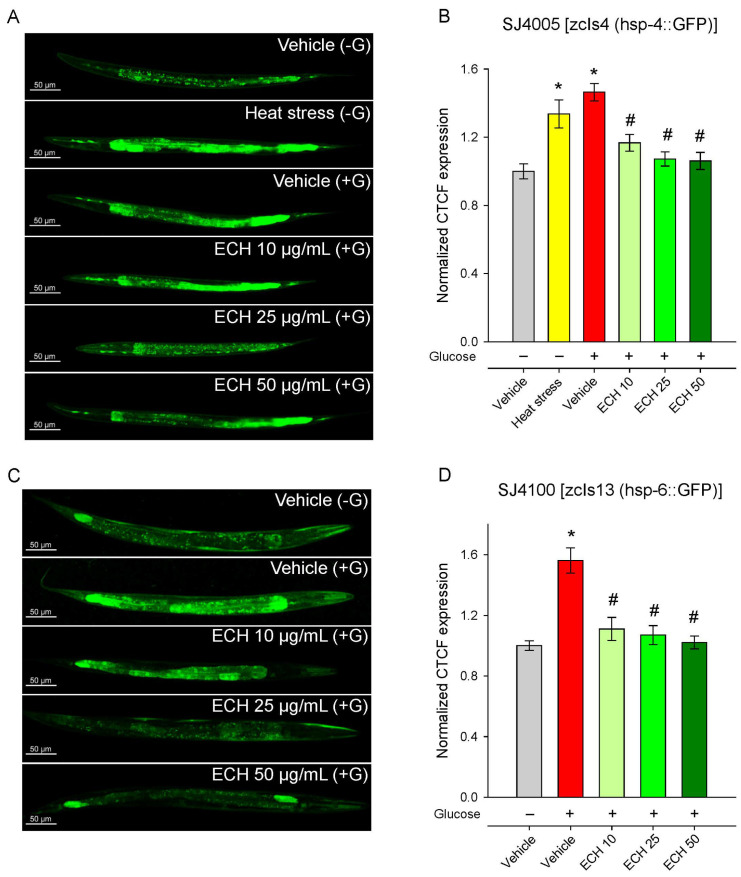
The extract of *E*. *tenuifolia* (ECH) attenuates glucose-induced activation of UPRmt and ER stress markers. Representative confocal images and CTCF, normalized to the vehicle (−G) group of (**A**,**B**) SJ4005 [zcIs4 (hsp-4::GFP)] and (**C**,**D**) SJ4100 [zcIs13 (hsp-6::GFP)] transgenic worms. Animals were treated for 24 h with 10, 25, or 50 μg/mL *E*. *tenuifolia* extract or vehicle (−G, +G). For the SJ4005 strain, a brief heat stress was used (37 °C for 5 min) as a positive control group for *hsp-4* expression. Data are presented as mean ± SEM, *n* = 60; * *p* < 0.05 compared to vehicle (−G), and # *p* < 0.05 compared to vehicle (+G); ANOVA on ranks.

Glucose supplementation induced a strong upregulation of *hsp-4*, reflecting activation of the ER stress response, likely due to glucose-induced metabolic or proteotoxic imbalance. Supplementation with *E. tenuifolia* extract dose-dependently suppressed *hsp-4* activation, restoring expression to baseline (Vehicle −G) levels (Figure 6A,B). This normalization suggests that the extract either prevents the accumulation of misfolded proteins under metabolic stress or enhances their clearance, thereby reducing ER burden.

Consistent with previous observations [49,50], exposure to 2% glucose markedly increased hsp-6::GFP fluorescence, indicating activation of the mitochondrial unfolded protein response (Figure 6C,D). The treatment with *E. tenuifolia* extract significantly reduced *hsp-6* expression in a dose-dependent manner (Figure 6C,D), with all concentrations (10, 25, and 50 μg/mL) lowering fluorescence to levels comparable to the control group without glucose (Vehicle −G). These findings suggest that the extract effectively alleviates glucose-induced mitochondrial stress.

Together, these results indicate that *E. tenuifolia* extract mitigates both mitochondrial and ER stress triggered by excess glucose, potentially contributing to the improved mitochondrial function and metabolic homeostasis observed in other assays.

## 4. Discussion

Poor treatment adherence; demographic aging; and the increasing prevalence of obesity, diabetes, and chronic kidney disease continue to limit the overall success of current cardiovascular therapies [51,52,53]. On the other hand, some interventions for age-related diseases, such as anti-neoplastic drugs, have been reported to induce cardiovascular toxicity [54,55]. Consequently, despite the availability of established therapies for cardiovascular diseases, there remains a need to explore complementary or alternative approaches targeting fundamental mechanisms that underlie cardiometabolic and age-related disorders. Mitochondrial dysfunction, impaired bioenergetics, and chronic metabolic stress are increasingly recognized as shared pathophysiological drivers of cardiovascular diseases. Therefore, strategies aimed at modulating mitochondrial function and improving cellular resilience to metabolic stress may offer benefits to both healthspan and disease prevention.

Natural products have long been recognized for their therapeutic potential, yet many remain underexplored in the context of human health. The Apiaceae family is particularly rich in species with both medicinal and nutritious properties [56]. Specifically, plants of the *Echinophora* genus have been reported to possess diverse pharmacological properties, including antibacterial, antifungal, anticancer, cytoprotective, and anti-inflammatory [2]. Recent studies have provided preliminary insights into the essential oil composition and biological activity of *E. tenuifolia* subsp. *sibthorpiana* [2,5]. However, *E. tenuifolia* extract has been poorly characterized in terms of both phytochemical profile and biological activity. To address this, we evaluated the chemical profile of the extract using NMR and HPLC-PDA. As a result, several well-characterized bioactive compounds were identified in the extract, including ferulic acid, caffeic acid, and chlorogenic acid, with rutin being the most abundant. These results confirm the previously reported phenolic profile of *E. tenuifolia*, with rutin being the most abundant flavonoid identified in the extract [57].

Several constituents identified in the extract have been associated with mitochondrial homeostasis and metabolic regulation. Rutin, for example, exhibits anti-inflammatory, anticancer, and antioxidant properties and has been proposed as a potential anti-ulcer agent, partly through inhibition of protein disulfide isomerase (PDI) [58]. Ferulic acid has been linked with attenuation of metabolic dysfunction and oxidative stress, partly through regulation of mitochondrial dynamics and antioxidant pathways [59]. In a zebrafish model of alcoholic fatty liver disease, rutin has been shown to reverse steatosis and restore mitochondrial dynamics, while in obese mice it reduced cognitive deficits [60].

In our study, the *E. tenuifolia* extract enhanced survival upon paraquat-induced oxidative stress at two distinct time points during the worms’ lifespan, suggesting improved stress resilience. This effect is consistent with a potential modulation of antioxidant defense mechanisms and mitochondrial function. However, direct measurements of intracellular ROS levels, ATP content, lipid peroxidation markers, or antioxidant enzyme expression were not performed, and therefore, the precise redox-related mechanisms remain to be clarified. Future studies incorporating these endpoints will be necessary to better define the extract’s impact on cellular oxidative status and bioenergetics.

In regard to thermotolerance, *E. tenuifolia* extract improved resilience to heat stress in young worms (day 5), as well as in aged (day 10) *C. elegans*, suggesting the involvement of proteostasis and stress-response networks following treatment. In this context, rutin has been reported to exert neuroprotective activity in models of Alzheimer’s and Parkinson’s disease, primarily through modulation of inflammatory signaling and antioxidant defense [61]. Regarding functional outcomes, the extract enhanced locomotor activity in young worms; however, locomotor improvements were absent in aged worms, and lifespan was not significantly extended. This distinction highlights that the extract primarily modulates functional and metabolic aspects of aging, consistent with our objective of evaluating its impact on healthspan and metabolic regulation [62].

Some of the constituents of the extract have been reported to modulate lipid metabolism and mitochondrial function. Rutin, for example, has been shown to decrease the atherogenic index and reduce body weight through modulation of AMP-activated protein kinase (AMPK), while simultaneously increasing mtDNA content and promoting mitochondrial biogenesis via ER stress modulation [63,64]. Chlorogenic acid, on the other hand, has been reported to regulate mitophagy through the PINK1/Parkin signaling pathway [65,66] and to ameliorate obesity-associated metabolic disturbances by improving lipid metabolism and mitochondrial quality control [67,68].

Consistent with these observations, *E. tenuifolia* extract supplementation reduced lipid accumulation in glucose-fed *C. elegans* and was associated with an improvement in _Δ_Ψm under glucose-induced stress. Notably, mitochondrial mass remained unchanged, suggesting that the extract does not promote mitochondrial proliferation but may influence the functional efficiency of existing mitochondria. However, the interpretation of increased _Δ_Ψm requires caution, as mitochondrial hyperpolarization can also occur under stress conditions and may reflect altered redox status rather than enhanced mitochondrial efficiency [69]. Furthermore, in the absence of complementary ATP production or ROS measurements, conclusions regarding improved mitochondrial performance remain indirect. Additional studies will, therefore, be necessary to clarify whether the observed changes in _Δ_Ψm are due to enhanced bioenergetics efficiency or other mitochondria-related adaptive mechanisms.

The extract significantly reduced the expression of *hsp-4* and *hsp-6*, restoring both to baseline levels in glucose-exposed worms. Given that these chaperones are central components of the ER stress response and UPRmt, respectively, this finding indicates that *E. tenuifolia* extract alleviates proteotoxic stress across both organelles. This is particularly relevant in the context of glucose-induced metabolic dysfunction, where sustained ER and mitochondrial stress contribute to impaired bioenergetics, lipid accumulation, and reduced cellular resilience. This observation is consistent with the chemically complex nature of the extract, which is enriched in phenolic compounds capable of interacting with multiple stress-response pathways. Rather than acting on a single target, the extract appears to modulate integrated stress-response networks involved in mitochondrial quality control. Indeed, caffeic and chlorogenic acids have been associated with the regulation of ER stress and AMPK-dependent metabolic adaptation [70,71,72], while rosmarinic acid has been reported to preserve mitochondrial integrity through mechanisms linked to the UPRmt [73]. Within this framework, the suppression of *hsp-6* expression observed in our study may reflect a reduced requirement for mitochondrial stress compensation, suggesting an overall improvement in mitochondrial proteostasis. This interpretation is further supported by the restoration of mitochondrial membrane potential and the reduction in lipid accumulation under glucose challenge, both of which are tightly coupled to mitochondrial efficiency and metabolic flexibility. Importantly, these effects occurred in the absence of changes in mitochondrial mass, indicating that the extract primarily influences mitochondrial function rather than biogenesis. This distinction highlights a potential shift toward improved mitochondrial quality rather than quantity, a mechanism increasingly recognized as a key determinant of healthspan [35]. Nevertheless, these interpretations remain indirect and would benefit from additional mechanistic investigations to clarify the precise signaling pathways involved.

The present study is limited to several aspects of mitochondrial dysfunction and metabolic dysregulation assessed in *C. elegans*. Nevertheless, due to the lack of bioactivity-guided fractionation and validation using isolated compounds, the relative contribution of individual constituents remains unresolved. While our findings reveal the beneficial effects of the extract on mitochondrial performance, stress resistance, and ER- and UPRmt-related chaperones, mechanistic conclusions remain limited. Further detailed mechanistic studies are needed to clarify the extract’s role in age-associated decline and its interactions with stress-response pathways. Additionally, the results should be interpreted with caution since plant extracts may exhibit variability in composition depending on factors such as harvesting conditions and extraction methods, which may limit the consistency of outcomes across different batches or studies.

## 5. Conclusions

Collectively, our findings support a model in which *E. tenuifolia* extract enhances metabolic resilience by modulating mitochondrial and ER stress signaling, thereby mitigating the detrimental effects of nutrient excess. Here, we hypothesize that the metabolism- and mitochondria-related beneficial effects of *E*. *tenuifolia* extract are likely associated with its phenolic-rich composition and the coordinated actions of multiple constituents, several of which have been reported to exert similar biological activities when tested individually. Thus, future studies examining the effects of isolated constituents, as well as potential synergistic interactions within the extract, will be necessary to clarify the role of individual compounds in the observed outcomes.

## Figures and Tables

**Table 1:** Mobile phase composition: A (0.1% formic acid in water), B (methanol), and C (acetonitrile).

Time (min)	A (%)	B (%)	C (%)
0	75	5	20
15	60	40	0
24	50	50	0
40	50	50	0
60	20	80	0
70	75	5	20

## Data Availability

The original contributions presented in this study are included in the article and Supplementary Materials. Further inquiries can be directed to the corresponding authors.

## Supplementary Materials

The following supporting information can be downloaded at https://www.mdpi.com/article/10.3390/antiox15030398/s1: Table S1: Chemical shifts (*δ*) and coupling constants (*J*) of the common primary and some secondary metabolites identified by their relevant ^1^H NMR spectra in *E. tenuifolia* extract compared with the literature. Table S2: System suitability parameters for the developed HPLC-PDA method. Table S3: Evaluation of linearity, limit of detection (LD), and limit of quantification (LQ) of the developed HPLC-PDA method for detection and quantification of phenolic acids and flavonoids. Table S4: Evaluation of accuracy of the developed HPLC-PDA method for detection and quantification of phenolic acids and flavonoids. Table S5: Evaluation of precision of the developed HPLC-PDA method for detection and quantification of phenolic acids and flavonoids. Table S6: Effect of *E*. *tenuifolia* extract treatment (10, 25, or 50 μg/mL) on mean and maximum survival (days) upon acute oxidative stress (50 mM paraquat) of *C*. *elegans* wild-type (N2) strain measured in two time points (days 5 and 10). Figure S1: HPLC-PDA chromatograms of standard mixture of flavonoids and phenolic acids at selected wavelengths.

## Abbreviations

The following abbreviations are used in this manuscript:

NGM	Nematode growth medium
ATP	Adenosine triphosphate
HPLC-PDA	High-performance liquid chromatography with photodiode array detection
NMR	Nuclear magnetic resonance
MVG	MitoView™ Green
TMRE	Tetramethylrhodamine ethyl ester
UPRmt	Mitochondrial unfolded protein response
CTCF	Corrected total cell fluorescence
ER	Endoplasmic reticulum
ΔΨm	Mitochondrial membrane potential
AMPK	AMP-activated protein kinase
ECH	*Echinophora tenuifolia* extract
CD_3_OD	Deuterated methanol
D_2_O	Deuterated water
