# Supplementary material for: Echinophora tenuifolia L. subsp. sibthorpiana Modulates Stress Response and Mitochondrial Quality Under Glucose Stress in Caenorhabditis elegans
Source: Antioxidants (Basel). 2026 Mar 21;15(3):398. doi: 10.3390/antiox15030398 (PMC13023430; doi:10.3390/antiox15030398)
Supplement: Supplementary file 1 [file antioxidants-15-00398-s001.zip › antioxidants-4187009-supplementary.pdf]

Supplementary information

Echinophora tenuifolia L. subsp. *sibthorpiana* modulates stress response and mitochondrial quality under glucose stress in *Caenorhabditis elegans*

Monika N. Todorova [1], Stanislav Dyankov [2,3], Martina S. Savova [1,4], Velislava Todorova [2,3], Milen I. Georgiev [1,4], Stanislava Ivanova [2,3,*]

[1] Laboratory of Metabolomics, Institute of Microbiology, Bulgarian Academy of Sciences, 4000 Plovdiv, Bulgaria

[2] Department of Pharmacognosy and Pharmaceutical Chemistry, Faculty of Pharmacy, Medical University of Plovdiv, 4002 Plovdiv, Bulgaria

[3] Research Institute, Medical University of Plovdiv, 4002 Plovdiv, Bulgaria

[4] Department of Plant Cell Biotechnology, Center of Plant Systems Biology and Biotechnology, 4000 Plovdiv, Bulgaria

***Corresponding author:**

E-mail address: **stanislava.ivanova@mu-plovdiv.bg (Stanislava Ivanova)**

Supplementary Tables

Table S1. Chemical shifts (*δ*) and coupling constants (*J*) of the common primary and some secondary metabolites identified by their relevant ^{1}H NMR spectra in *E. tenuifolia* extract compared with literature.

**Metabolite**	**Chemical shift (*δ*, ppm)**	**Coupling constant (*J*, Hz)**
α-Glucose	5.19	(d, *J* = 3.8)
β-Glucose	4.59	(d, *J* = 7.9)
Sucrose	5.41/4.18	(d, *J* = 3.9)/(d, *J* = 8.7)
Alanine	1.49	(d, *J* = 7.3)
Valine	1.01/1.06	(d, *J* = 7.0)/(d, *J* = 7.0)
Threonine	1.33	(d, *J* = 6.8)
Formic acid	8.47	(s)
Malic acid	2.38/2.68/4.28	(dd, *J* = 15, 9.9)/(dd, *J* = 15.5, 3.3)/(dd, *J* = 9.6, 3.19)
Pyruvic acid	2.43	(s)
γ-Amino-butyrate (GABA)	1.94/2.30/3.01	(m)/(t, J = 7.5)/(t, J = 7.4)
Ferulic acid	6.36	(d, *J* = 15.8)
Rutin	7.67/7.61/6.98/6.51/6.30/4.99/4.54/ 3.80/3.62/3.54/3.52/3.51/3.44/3.40/ 3.38/3.37/3.31/1.09	(d, *J* = 8.7)/(dd, *J* = 8.5, 2)/(d, *J* = 8.5) /(d, *J* = 2)/(d, *J* = 2)/(d, *J* = 7.89)/(d, *J* = 1.49)/(dd*)/(dd*)/(d, *J* = 7.5)/(dd, *J* = 9.5, 3.5)/ (dd*)/(m*)/(m)/ (d, *J* = 9)/ (d, *J* = 9)/ (d, *J* = d*)/(d, *J* = 6)

*overlapping region

Table S2. System suitability parameters for the developed HPLC-PDA method.

**Compound**	**Selectivity factor**	**Resolution**	**Number of theoretical plates**	**Asymmetry factor**
Chlorogenic acid	–	–	8590	1.07
Catechin	1.27	1.79	9480	0.92
Caffeic acid	1.88	6.67	10783	1.08
Rutin	1.55	5.49	6832	1.06

p-Coumaric acid	1.37	3.05	9978	1.08
Ferulic acid	1.24	14.65	10746	1.04
Hesperidin	1.43	6.09	6724	1.04
Rosmarinic acid	1.18	3.23	14536	1.02
Salicylic acid	1.16	3.52	12897	1.04
Quercetin	1.66	18.56	62819	1.11
Luteolin	1.09	4.82	49986	1.08
Kaempferol	1.27	11.12	36653	1.11
Apigenin	1.05	2.19	33482	1.07
Casticin	1.30	17.43	264355	1.06
Acacetin	1.08	10.59	354986	1.09

Table S3. Evaluation of linearity, limit of detection (LD), and limit of quantification (LQ) of the developed HPLC-PDA method for detection and quantification of phenolic acids and flavonoids.

Compound	Linear regression line	R^2	LD (μg/mL)	LQ (μg/mL)
Chlorogenic acid	y = 41832x + 107954	0.9999	1.13	3.41
Catechin	y = 14060x - 6028,1	0.9999	0.96	2.90
Caffeic acid	y = 118421x - 69302	0.9999	1.18	3.57
Rutin	y = 41585x + 8179	0.9999	1.27	3.86
p-Coumaric acid	y = 143605x + 5083,5	0.9999	0.84	2.54
Ferulic acid	y = 97780x - 18558	0.9999	0.94	2.85
Hesperidin	y = 25762x - 13528	0.9999	0.80	2.43
Rosmarinic acid	y = 59765x - 32572	0.9999	0.79	2.39
Salicylic acid	y = 61091x - 43374	0.9999	1.19	3.59
Quercetin	y = 76898x - 45411	0.9999	1.07	3.23
Luteolin	y = 77836x - 21524	0.9999	0.85	2.57
Kaempferol	y = 72582x - 34084	0.9999	0.96	2.96
Apigenin	y = 101010x - 102720	0.9999	1.02	3.09
Casticin	y = 69468x - 36057	0.9999	1.09	3.31
Acacetin	y = 73470x - 87956	0.9999	1.07	3.25

Table S4. Evaluation of accuracy of the developed HPLC-PDA method for detection and quantification of phenolic acids and flavonoids.

Concentration (µg/mL)	Mean (µg/mL ± SD)	Recovery %	CV %
**Chlorogenic acid**			
50	50.02 ± 0.01	100.04	0.03
25	25.03 ± 0.05	100.13	0.19
10	10.02 ± 0.03	100.22	0.25
**Catechin**			
50	49.99 ± 0.08	99.99	0.16
25	24.99 ± 0.08	99.99	0.34
10	10.07 ± 0.01	100.72	0.14
**Caffeic acid**			
50	49.75 ± 0.01	99.50	0.02
25	25.52 ± 0.01	101.59	0.79
10	10.17 ± 0.14	101.75	2.45
**Rutin**			
50	50.79 ± 0.87	101.59	1.73
25	25.39 ± 0.04	101.57	0.14
10	10.04 ± 0.02	100.44	0.15
***p*-Coumaric acid**			
50	50.17 ± 0.04	100.34	0.07
25	25.11 ± 0.01	100.42	0.03
10	10.15 ± 0.06	101.54	0.60
**Ferulic acid**			
50	50.10 ± 0.07	100.20	0.14
25	25.21 ± 0.02	100.84	0.07
10	10.22 ± 0.03	102.24	0.34
**Hesperidin**			
50	50.14 ± 0.02	100.28	0.05
25	25.38 ± 0.02	101.50	0.07
10	10.02 ± 0.02	100.20	0.21
**Rosmarinic acid**			
50	49.95 ± 0.02	99.90	0.05
25	24.98 ± 0.01	99.93	0.02
10	10.07 ± 0.01	100.75	0.09
**Salicylic acid**			
50	50.44 ± 0.02	100.88	0.04
25	25.17 ± 0.02	100.70	0.07

10	10.04 ± 0.01	100.37	0.07
**Quercetin**			
50	50.49 ± 0.02	100.99	0.04
25	25.10 ± 0.02	100.42	0.07
10	10.01 ± 0.01	100.09	0.09
**Luteolin**			
50	50.18 ± 0.01	100.35	0.02
25	25.24 ± 0.01	100.96	0.02
10	10.06 ± 0.01	100.60	0.08
**Kaempferol**			
50	50.14 ± 0.01	100.28	0.02
25	25.11 ± 0.01	100.48	0.05
10	10.08 ± 0.01	100.77	0.06
**Apigenin**			
50	50.04 ± 0.01	100.07	0.01
25	25.05 ± 0.01	100.20	0.04
10	10.09 ± 0.01	100.94	0.04
**Casticin**			
50	50.24 ± 0.09	100.48	0.17
25	25.06 ± 0.02	100.25	0.10
10	10.04 ± 0.01	100.38	0.08
**Acacetin**			
50	50.02 ± 0.01	100.04	0.01
25	25.05 ± 0.05	100.22	0.20
10	10.06 ± 0.01	100.55	0.06

Table S5. Evaluation of precision of the developed HPLC-PDA method for detection and quantification of phenolic acids and flavonoids.

Concentration (µg/mL)	Intra-Day Precision			Inter-Day Precision		
	Mean (µg/mL ± SD)	SEM	CV %	Mean (µg/mL ± SD)	SEM	CV %
**Chlorogenic acid**						
50	50.03 ± 0.01	0.01	0.02	50.02 ± 0.03	0.02	0.06
25	24.70 ± 0.01	0.01	0.05	25.20 ± 0.14	0.08	0.56
10	10.06 ± 0.01	0.01	0.14	10.04 ± 0.06	0.03	0.59
**Catechin**						
50	49.58 ± 0.01	0.01	0.01	49.68 ± 0.14	0.08	0.30
25	24.87 ± 0.02	0.01	0.06	25.05 ± 0.03	0.02	0.13

10	10.13 ± 0.01	0.01	0.11	10.13 ± 0.02	0.02	0.26
**Caffeic acid**						
50	49.75 ± 0.01	0.01	0.04	50.01 ± 0.08	0.05	0.16
25	25.52 ± 0.01	0.01	0.03	26.00 ± 0.05	0.03	0.20
10	10.47 ± 0.01	0.01	0.11	10.15 ± 0.10	0.05	0.97
**Rutin**						
50	51.31 ± 0.02	0.01	0.04	51.02 ± 0.50	0.26	0.88
25	25.60 ± 0.02	0.01	0.08	25.56 ± 0.04	0.02	0.15
10	10.09 ± 0.05	0.03	0.54	10.07 ± 0.02	0.01	0.23
***p*-Coumaric acid**						
50	50.19 ± 0.05	0.03	0.09	50.12 ± 0.05	0.03	0.10
25	25.01 ± 0.01	0.01	0.03	25.11 ± 0.07	0.04	0.27
10	10.13 ± 0.01	0.01	0.07	10.16 ± 0.01	0.01	0.15
**Ferulic acid**						
50	50.02 ± 0.01	0.01	0.09	50.13 ± 0.08	0.04	0.17
25	25.23 ± 0.02	0.01	0.08	25.18 ± 0.03	0.02	0.11
10	10.29 ± 0.01	0.01	0.02	10.31 ± 0.01	0.03	0.03
**Hesperidin**						
50	50.12 ± 0.04	0.02	0.09	50.03 ± 0.09	0.05	0.17
25	25.37 ± 0.03	0.02	0.13	25.35 ± 0.06	0.04	0.25
10	10.03 ± 0.02	0.01	0.22	10.04 ± 0.03	0.02	0.31
**Rosmarinic acid**						
50	49.97 ± 0.06	0.03	0.11	50.19 ± 0.30	0.17	0.60
25	25.10 ± 0.02	0.01	0.08	25.06 ± 0.03	0.02	0.14
10	10.08 ± 0.01	0.01	0.02	10.07 ± 0.01	0.01	0.13
**Salicylic acid**						
50	50.49 ± 0.02	0.01	0.04	50.38 ± 0.04	0.02	0.08
25	25.23 ± 0.02	0.01	0.10	25.00 ± 0.04	0.02	0.17
10	10.03 ± 0.01	0.01	0.10	10.00 ± 0.02	0.01	0.16
**Quercetin**						
50	50.50 ± 0.02	0.01	0.04	50.52 ± 0.03	0.02	0.06
25	25.14 ± 0.01	0.01	0.04	25.08 ± 0.05	0.03	0.21
10	10.01 ± 0.01	0.01	0.07	10.01 ± 0.01	0.01	0.07
**Luteolin**						
50	50.22 ± 0.02	0.01	0.03	50.21 ± 0.02	0.01	0.06
25	25.24 ± 0.01	0.01	0.04	25.25 ± 0.01	0.01	0.03
10	10.08 ± 0.01	0.01	0.06	10.09 ± 0.01	0.01	0.11
**Kaempferol**						
50	50.17 ± 0.02	0.01	0.04	50.16 ± 0.03	0.02	0.07

25	25.10 ± 0.01	0.01	0.06	25.06 ± 0.05	0.02	0.19
10	10.05 ± 0.01	0.01	0.11	10.05 ± 0.04	0.02	0.40
			**Apigenin**			
50	50.07 ± 0.03	0.02	0.06	50.08 ± 0.06	0.04	0.13
25	25.03 ± 0.04	0.02	0.16	25.25 ± 0.16	0.10	0.66
10	10.08 ± 0.01	0.01	0.07	10.09 ± 0.02	0.01	0.17
			**Casticin**			
50	50.22 ± 0.03	0.02	0.05	50.32 ± 0.11	0.06	0.22
25	25.09 ± 0.02	0.01	0.06	25.03 ± 0.09	0.05	0.34
10	10.02 ± 0.01	0.01	0.05	10.02 ± 0.02	0.01	0.16
			**Acacetin**			
50	50.05 ± 0.02	0.01	0.05	49.94 ± 0.06	0.03	0.12
25	25.10 ± 0.01	0.01	0.06	25.08 ± 0.07	0.04	0.27
10	10.05 ± 0.01	0.01	0.06	10.05 ± 0.02	0.01	0.20

Table S6. Effect of *E. tenuifolia* extract (ECH) treatment (10, 25, or 50 μg/mL) on mean and maximum survival (days) and mean survival increase (%) upon acute oxidative stress (50 mM paraquat) of *C. elegans* wild-type (N2) strain measured in two time points (day 5 and 10).

Time point	Treatment		Mean survival (SEM)	Mean survival increase (%)	Maximum survival (SEM)
day 5	Vehicle		2.16 (0.12)	–	4 (0)
	ECH	10	2.66 (0.13)	23.20	4 (0)
		25	3.18 (0.15)	47.42	5 (0)
		50	3.67 (0.17)	70.10	6 (0)
day 10	Vehicle		1.57 (0.08)	–	3 (0)
	ECH	10	1.59 (0.08)	1.42	3 (0)
		25	1.86 (0.09)	18.44	3 (0)
		50	1.98 (0.09)	26.24	3 (0)

Supplementary Figures

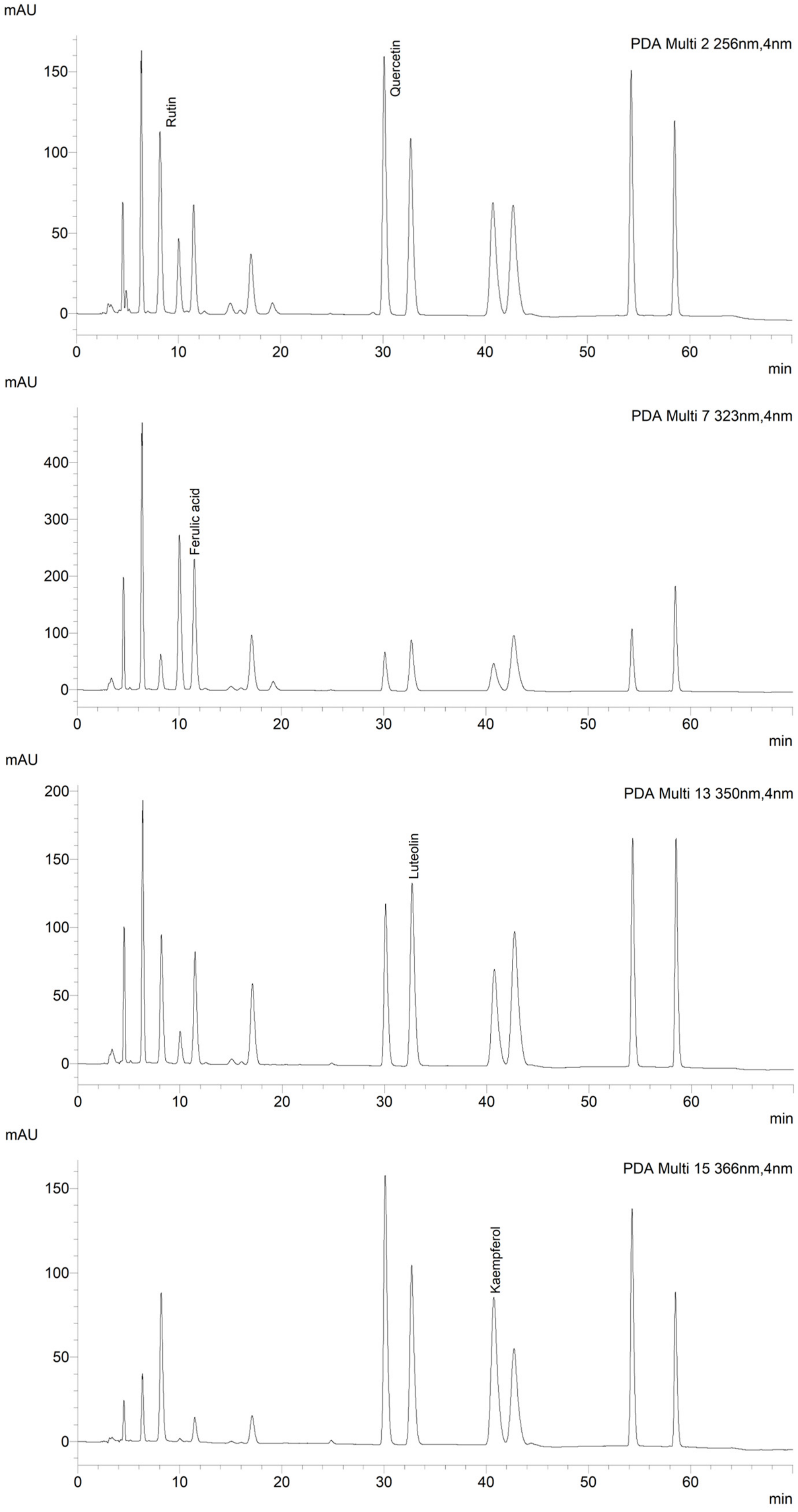

Figure S1. HPLC-PDA chromatograms of standard mixture of flavonoids and phenolic acids at selected wavelengths.
